# Curbing Inflammation in Burn Patients

**DOI:** 10.1155/2013/715645

**Published:** 2013-05-20

**Authors:** Jayme A. Farina, Marina Junqueira Rosique, Rodrigo G. Rosique

**Affiliations:** Department of Surgery and Anatomy, Division of Plastic Surgery, School of Medicine of Ribeirão Preto-SP, University of São Paulo, Avenida Bandeirantes 3900, 9.°andar, 14048-900 Ribeirão Preto SP, Brazil

## Abstract

Patients who suffer from severe burns develop metabolic imbalances and systemic inflammatory response syndrome (SIRS) which can result in multiple organ failure and death. Research aimed at reducing the inflammatory process has yielded new insight into burn injury therapies. In this review, we discuss strategies used to curb inflammation in burn injuries and note that further studies with high quality evidence are necessary.

## 1. Introduction

Trauma resulting from severe burn injury triggers a systemic inflammatory response syndrome (SIRS) and serious metabolic disturbances. One of the best known systemic manifestations observed in the first hours after a major burn injuries is related to increased systemic capillary permeability with protein leakage into the interstitial space, generalized edema, and a tendency toward hypovolemic shock. Adequate fluid replacement is mandatory in the first hours after a traumatic burn. However, in burn patients, other systemic disorders are also accompanied by SIRS such as cardiac dysfunction, acute respiratory distress syndrome, acute renal failure, increased intestinal permeability resulting in bacterial translocation, hypermetabolism, hypercatabolism, and sepsis [1–4]. These intense disruptions in body's homeostatic balance may result in multiple organ failure and death. Therefore, research seeking new mechanisms by which to attenuate inflammation after severe burn injury is needed. In this review, we address and discuss the available options.

## 2. Burns and Inflammation

Burn injury induces global changes to the entire immune system resulting in suppressed immune function and increased susceptibility to infection. This immunopathological response can contribute to the development of SIRS and subsequent multiple organ failure. Patients with severe burns are more likely to die from sepsis due to the massive release of inflammatory mediators from the burn wounds. Total body surface area (BSA) involved and smoke inhalation are predictors of death. Each one percent increase in total body surface area burned was associated with a six percent increase in mortality risk. Also, the presence of smoke inhalation increased mortality risk by ninefold [5]. In addition, the depth of the burn also affects mortality risk as full thickness burns have a poorer prognosis compared to partial thickness.

Nevertheless, the systemic disorders observed in the first hours after a severe burn injury are related to increased systemic capillary permeability with protein leakage and a tendency toward hypovolemic shock. Burns greater than 10% BSA in children or 15% BSA in adults are potentially life-threatening injuries (because of the risk of hypovolemic shock) and should be treated with formal fluid resuscitation and monitoring in a burn unit [6]. Hence, adequate fluid replacement is mandatory in the first 24 hours after the severe burn trauma minimizing the possibility of hypovolemia and early renal insufficiency. The patient with extensive burns will undergo surgery only after appropriate fluid resuscitation, which usually occurs after 48–72 hours. However, fluid resuscitation must be undertaken judiciously as excess fluids may worsen the prognosis of burn patients and care must always be present to restrict the supply of liquid to only what is necessary.

## 3. Volemic Resuscitation and Inflammation

### 3.1. Fluid-Restrictive Strategies

Despite the convenience of using formulas as an initial guide for fluid replacement (i.e., the Parkland formula: 4 mL∗weight  (kg)∗%  BSA), it is difficult to carry out fine adjustments in fluid delivery to the severely burned patient in practice. Commonly, there is a tendency to administer an oversupply of fluid (fluid creep) [7–9]. But what degree of excess crystalloid hydration leads to systemic complications after burns? Increasing evidence has demonstrated that aggressive crystalloid-based resuscitation strategies are associated with cardiac and pulmonary complications, gastrointestinal dysmotility, coagulation disturbances, and immunological and inflammatory mediator dysfunction. Numerous investigators have evaluated potential risk factors for developing abdominal compartment syndrome and have universally noted the excessive use of crystalloids as the primary determinant [10–12]. In our experience, we have observed that elevated levels of creatinine associated with disturbances in renal function occur concurrently with the initial signs of abdominal compartment syndrome, even without evidence of sepsis. After careful fluid restriction and diuresis induction, generalized reduction of edema is observed along with normalization of renal function. Also, disturbances in cell volume disrupt numerous regulatory mechanisms responsible for keeping the inflammatory cascade under control [10, 11].

In the last decade, our burn center staff has preferred the use of the formula: 3 mL∗weight  (kg)∗% BSA of crystalloid infusion instead of the Parkland formula in the first 24 hours after burn injury. Using our formula, we have observed only minor amounts of general edema in the first days after extensive burn trauma, with consequent reduced morbidity and faster recovery. Our findings, “data not shown,” have been shared by other authors [10, 13]. Fluid-restrictive strategies have been associated with a decreased frequency of and shorter time to recovery from acute respiratory distress syndrome and trends toward shorter lengths of stay and lower mortality [10, 13]. The proper control of liquids provides the ability to perform surgery earlier in patients with severe burns, thus accelerating healing.

## 4. Inflammation Related to the Wound 

The lipid protein complex (LPC) released from burnt skin is responsible for the profound immune suppression associated with major cutaneous burns [14, 15]. Thermal injury represents a pathophysiological condition in which hyperactive macrophages are primed to stimulate the downregulation or upregulation of certain inflammatory cytokines [16–18]. Abnormal levels of proinflammatory mediators, such as tumor necrosis factor alpha (TNF-*α*), interleukin-1b (IL-1b), interleukin-6 (IL-6), interleukin-8 (IL-8), and interleukin-10 (IL-10), have been reported both systemically and locally in burn patients. It has been proposed that there is a genetic influence on cytokine production and susceptibility to sepsis. A recent and interesting study indicates that genetically determined individual differences in IL-10 production might influence the susceptibility to septic complications in burned patients [19].

Furthermore, attempts to understand immune responses in different burn depths may produce knowledge about the pathophysiology of major burns [20]. Sakallioglu et al. showed that the circulating levels of the proinflammatory cytokines, IL-6 and interferon-gamma (IFN-*γ*), were higher in rats with full thickness burns as compared to rats with only partial thickness burns, one hour after burn injury. The authors suggested that early elevation of IL-6 and IFN-*γ* can prolong inflammation in full-thickness burns [20]. 

Thus, the rationale for early excision of burns is the decrease in release of inflammatory mediators and bacterial colonization of wounds. This, in turn, can attenuate SIRS and reduce the occurrence of metabolic derangements, sepsis, and multiorgan failure [21]. When performed early, excision and immediate wound closure has been shown to improve survival and decrease length of hospital stay in burn patients [21].

### 4.1. Escharectomy

The concept of early excision of burned tissue was developed by Janzekovic in 1970, when she enumerated several advantages of this concept including removal of tissue before bacterial colonization (from 3 to 5 days after burn trauma); patients in better physical condition; improved scar quality; fewer contractures; shorter hospital stay; fewer dressing changes [22]. Skin burns result in an intense inflammatory response; thus, it has been proposed that early surgical removal of burned tissue might limit the increased production of inflammatory mediators.

In rats, escharectomy while the animal is still in shock can inhibit the overexpression of both early and late inflammatory mediators and maintain the balance of pro-/anti-inflammatory responses, thereby improving multiple organ function following severe burns [23]. Additionally, in humans, escharectomy has an immunomodulatory effect on the inflammatory mediators and reduces insulin resistance induced by major burns [24]. Moreover, both thermal injury and escharectomy induce endothelial progenitor cells (EPCs) production. EPCs migrate to sites of neovascularization in response to mediators contributing to wound healing [25]. Nevertheless, Han et al. showed a limited immunomodulatory effect of escharectomy on the inflammatory mediators in systemic inflammatory responses in burns which varied based on the extent and timing of surgery [26]. They reasoned that, in their study, only the samples taken from survivors were used for the final analysis and perhaps those patients experienced lower levels of postinjury stress.

In 1992, our burn center, Hospital das Clínicas da Faculdade de Medicina de Ribeirão Preto da Universidade de São Paulo-HC-FMRP-USP-Brazil, changed the standard of care in severe burns from delaying surgery until after eschar separation is observed to early excision and grafting; as a result, we observed a reduction in the mortality rate from 12% to less than 5% (Table 1).

Although early surgery is well established as essential in reducing inflammation and promoting better wound healing, other studies have reported the use of drugs to assist the modulation of inflammation after extensive and deep burns.

### 4.2. Antibodies Targeting Growth Factors

Recently, Sun et al. [27] investigated the effects of topically inhibiting two of these proinflammatory mediators: TNF-*α* and IL-6. They concluded that topical application of antibodies targeting TNF-*α* was effective in preventing progression of necrosis in a partial-thickness rat burn model. However, there is still clearly an increased risk for infection at injury sites and the dose-response behavior will be critical for evaluating the potential clinical efficacy of these materials.

### 4.3. Glucan Phosphate

Glucan phosphate (glucan), a soluble polysaccharide immunomodulator, is a modified water-soluble [beta]-1,3-linked glucose polymer derived from the cell wall of the yeast, *Saccharomyces cerevisiae* [28, 29]. Glucan phosphate treatment attenuates burn-induced inflammation and increases resistance to *Pseudomonas aeruginosa* burn wound infection in an experimental model of burn injury. Lyuksutova et al. [30] observed that intraperitoneal injection of glucan phosphate (40 mg/kg) improved survival in mice exposed to *Pseudomonas aeruginosa* in mice with 35% total BSA thermal injuries. Improved survival correlated with lower bacterial burden in the burn wound, attenuated production of proinflammatory cytokines, and enhanced production of Th1 (T helper 1) cytokines. These studies show that glucan phosphate treatment attenuates burn-induced inflammation and increases resistance to *Pseudomonas aeruginosa* burn wound infection in an experimental model of burn injury [30]. 

### 4.4. Ulinastatin

Ulinastatin is a protease inhibitor obtained from human urine; it has been shown to have anti-inflammatory effect by suppressing the production of proinflammatory cytokines [31]. Luo et al. found that treatment with ulinastatin (40,000 U/kg) could attenuate the systemic inflammatory response and visceral vasopermeability both *in vivo* and *in vitro* and may serve as a therapeutic agent for the prevention of a systemic inflammatory response and leakage of fluid into tissue after major burn injuries [32]. 

## 5. Inflammation and Coagulopathy

Coagulation and inflammation may interact during thermal injuries with major consequences for the pathogenesis of microvascular injury and subsequent multiple organ dysfunction or failure [33]; their interaction may also place patients at higher risk for development of septic complications [34]. The activation of inflammation and coagulation cascade in septic burn patients can ultimately lead to increased mortality [35]. The coagulopathy seen in burn patients is associated with the marked depletion of the endogenous regulators of the coagulation system [36–38]. Coagulation system dysfunction during the early postburn period is characterized by activation of procoagulation pathways, enhanced fibrinolytic activity, and impairment of natural anticoagulant activity. Both the thrombotic and fibrinolytic pathways are triggered proportionally to the extent of the burn [35, 36, 39, 40]. 

Treatment focused on the normalization of coagulation and the inhibition of systemic inflammation might have a positive impact on organ function and overall outcome in septic burn patients [34]. Molecular pathways contributing to inflammation-induced activation of coagulation and modulation of inflammation by coagulation factors have been reported in the literature over the last decade [35–38]. The activation of the thrombomodulin-protein C pathway plays a central role in the pathogenesis of acute traumatic coagulopathy [41]. Activated protein C (APC) is an important physiological anticoagulant derived from protein C by the action of the thrombomodulin-thrombin complex on endothelial cells [42]. Factor VIIa serves an important role both in the initiation of coagulation and in the activation of platelets [43]. Treatment strategies using antithrombin, protein C, and recombinant factor VIIa are based on early and continuous assessment of the bleeding and coagulation status of burn patients. The routine use of human recombinant activated protein C in burn patients with severe sepsis or septic shock is not recommended because there is a need for a large scale clinical trial to assess the benefits and harms of activated protein C in burn patients [34]. Administration of recombinant factor VIIa in acutely bleeding burn patients is recommended only if other (conventional) therapeutic options are not effective since the safety and the effectiveness of recombinant factor VIIa in burn patients have not been established in a randomized control trial [34]. 

Treatment of coagulation abnormalities is a challenge for burn specialists. Clear indications, efficacy, and the economic feasibility of the use of specific coagulation factors in burn patients should be targeted in clinical trials over the next few years.

## 6. Inflammation, Nutrition, and Hormones

Nutritional support plays an undisputed part in the treatment of critically ill patients. Enteral nutrition supplemented with arginine, for example, alters intestinal homogenates from a pro- to an anti-inflammatory profile in mice with large areas of burn injury [44]. Nutritional support must be individually tailored in terms of quantity and quality; thus, adapting the support to requirements appears as a priority of nutritional assessment [45].

Studies have shown that increased systemic retinol binding protein (RBP) levels are associated with insulin resistance (IR) and hyperinflammation in diabetic and obese patients. Increased RBP levels occurring after burn injury correlate with increased IR, inflammatory and catabolic responses, incidence of multiorgan failure, and mortality [46]. Insulin decreases mortality and prevents multiorgan failure in critically ill patients. Jeschke et al. reported that insulin administration decreased proinflammatory cytokines and proteins while increasing activation of an anti-inflammatory response in children with burn injuries [47]. 

SIRS is associated with a debilitating systemic hypercatabolic state, which is mediated by cytokines and chemokines [48]. Systemic inflammatory and hepatic acute-phase responses contribute to hypermetabolism, multiorgan failure, and mortality. 

One protein that has been studied in inflammation control is leptin. Leptin is a circulating hormone that regulates energy intake and expenditure, including appetite and metabolism. It is well established that leptin is involved in the regulation of inflammation [49–51]. Leptin can exert a direct effect on T cells and monocytes, causing the release of cytokines. It may also induce angiogenesis or influence angiogenic factors. Cytokines and leptin are increased in severely burned patients, including those cases associated with sepsis and those patients who ultimately do not survive their injuries while basic fibroblast growth factor (bFGF) and transforming growth factor alpha (TGF*α*) levels are lower in severe cases. These variations in cytokine levels may indicate impaired healing in severe burn injury patients which leads to their poorer prognosis. 

Leptin production is acutely increased during infection and inflammation, as a part of the host's acute phase response [52]. On the other hand, in a thermal injury model in rats, exogenous leptin reduced microscopic damage scores in the liver, stomach, colon, and kidney and also reduced death of mononuclear cells and granulocytes. It has been suggested that leptin may diminish burn-induced inflammation and associated multiple organ failure [53]. It is possible that endogenous elevation of leptin levels during burn injury is not sufficient to enhance healing and avoid organ damage. Leptin can be regarded as a novel treatment modality to diminish burn-induced inflammation, reduce postburn immune dysfunction, and enhance burn healing [54]. 

Another hormone that should be mentioned in inflammation in burns is cortisol. Although hypercortisolemia has been suggested as a primary hormonal mediator of whole-body catabolism following severe burn injury, it may not play a central role in the postburn hypermetabolic catabolic response [55].

## 7. Oxidative Stress and Inflammation

Oxidative stress has been documented in burn injuries in both animals [56] and humans [57]. Increased free radical levels can potentiate the clotting process, aid wound reepithelization, promote angiogenesis, and influence the bactericidal ability of neutrophils and macrophages [58]. However, the oxidants must be detoxified to prevent damage to the host cells [59], a process that requires a delicate balance between oxidants and antioxidants in biological systems [60].

A patient's vitamin status is directly involved in inflammation, antioxidant response, burn wound healing, and the expected immune responses [61]. Adjuvant administration of high-dose vitamin C during the first 24 h after thermal injury significantly reduced resuscitation fluid volume requirements, body weight gain, and wound edema in humans [62, 63]. Our group recently evaluated vitamin status as it related to inflammatory and oxidative stress markers in adult patients up to three days after thermal injury. This prospective study detected decreased serum levels of vitamin C in burn patients [64]. The low levels of vitamin C can be explained by augmented cutaneous loss of ascorbic acid. Moreover, large vitamin C expenditure may have taken place in extracellular compartments, neutralizing free radicals and aiding regeneration of vitamin E, which protects the cell from lipid peroxidation [65]. In this context, there is evidence of the clinical benefits of parenteral vitamin C administration in oxidative stress conditions [66].

### 7.1. Methylene Blue (MB): A Selective Inhibitor of Guanylate Cyclase and Nitric Oxide Synthase (NOS) Inhibitors

It is known that severe burns can be accompanied by vasoplegic syndrome (VS), a phenomenon which is characterized by persistent and diffuse vasodilation, hypotension, and low vascular resistance resulting in circulatory and respiratory failure. The decrease in systemic vascular resistance observed in VS is associated with excessive production of nitric oxide (NO). The plasma NO content is increased during the first hours after burn injury. It seems that the increased concentration of NO, combined with other biochemical phenomena of the systemic inflammatory response, leads to a widespread leakage of protein and intravascular fluid into the interstitial space, resulting in various degrees of edema and hypovolemia. Nitric oxide stimulates soluble guanylate cyclase to increase cyclic guanosine monophosphate (cGMP) production, leading to smooth muscle relaxation. The NO competitor, methylene blue (MB), which is an inhibitor of the soluble guanylate cyclase (sGC), has been proposed in the treatment of refractory cases of vasoplegia. We suggest MB as a viable, safe, and useful coadjuvant therapeutic tool to be used during fluid resuscitation [67, 68]. 

Another way to suppress vascular hyperpermeability is the administration of nitric oxide synthase (NOS) inhibitors [69]. However, NOS inhibitors are not currently in clinical use due to their lack of specificity, as they carry the consequent risk of generalized tissue necrosis. For these reasons, it seems more appropriate to use MB as a therapeutic agent in the aforementioned shock-related vasoplegic states. Methylene blue does not interfere with NOS and has played a longstanding beneficial role in many other clinical conditions. As a potent guanylate cyclase inhibitor, MP blocks the increase in cGMP levels and, consequently, prevents vascular smooth muscle NO endothelium-dependent relaxation [70]. We feel that a controlled randomized trial to assess MB as a viable coadjuvant therapeutic tool of fluid resuscitation in burn injury is a necessary next step.

## 8. Inflammation and Inhalation 

The intrinsic mechanisms by which inhalation injury contributes to elevated mortality are unclear. One hypothesis is that a massive influx of inflammatory mediators and cells (primarily neutrophils which can release mitochondrial DNA to the extracellular space on activation) results in direct injury to the airway, leading to production of secretions and airway obstruction with mucus and products of inflammatory cell breakdown including extracellular deoxyribonucleic acid [71, 72].

Joyner et al. [72] found markedly elevated DNA levels in airway secretions in children early after burn or inhalation injury. Elevated extracellular DNA levels are correlated with the presence of cytokine markers of inflammation and injury such as IL-6, IL-8, and TGF-*β*1. Potential sources of extracellular DNA in airway secretions include directly damaged epithelial cells and inflammatory cells responding to injury or infection [73]. Therefore, treatment with deoxyribonuclease (DNAse) has shown some clinical benefit in terms of improvement in airways obstruction [74, 75]. These studies raise the possibility of use DNAse for treatment after burn inhalation injury.

## 9. Other Possibilities

### 9.1. Mesenchymal Stem Cell (MSC)

In addition to the use of MSCs in regenerative research, it is known that these cells can also be used for modulation of inflammation. Bone-marrow-derived MSC allografts administered via intravenous transplantation have been shown to decrease proinflammatory cytokines, increase anti-inflammatory cytokines, decrease lung water mass fraction, ameliorate the systemic inflammatory response, and protect lung tissue in rabbits with smoke inhalation injury [76]. MSCs may also enhance burn wound healing and attenuate the immunosuppressant effects of the exacerbated inflammatory response and hypermetabolism in large burn injuries. Stem cell therapy may offer an additional means of mitigating burn hypercatabolism by preventing apoptosis of burned tissue. Investigation of the mechanism for this apoptotic arrest may reveal approaches toward slowing muscle breakdown associated with hypermetabolic response to burn [77, 78].

MSCs have a therapeutic benefit in burn-injured animals by providing anti-inflammatory as well as antiapoptotic effects. Human MSCs administered to the muscle of burned rats reduced infiltration of inflammatory cells into organs as kidney, lung, and liver. Interleukin-10 (IL-10) is one of the key cytokines with anti-inflammatory capacities; it has been demonstrated that MSCs can enhance the secretion of IL-10 by macrophages or dendritic cells. Furthermore, the activation of the AKT (V-murine thymoma viral oncogene homolog 1) signaling pathway in transplanted MSCs can provide an antiapoptotic effect in clinical settings of systemic inflammation [79].

### 9.2. Hyperbaric Oxygenation

In an experimental study in rats, treatment with hyperbaric oxygen accelerated the recovery from burn wounds and reduced the development of scars [80]. Rates of inflammation and fibrosis were lower hyperbaric-oxygen-treated animals as compared to controls. Hyperbaric oxygen maintains tissue viability by preventing microvascular tissue damage, reducing edema, and providing adequate oxygen to the damaged tissues. Hyperbaric treatment supports wound healing by reducing edema, improving microcirculation, decreasing the inflammatory response, and accelerating epithelialization. It may also reduce the progressive systemic effects of burns. Nevertheless, there is no consensus in the literature regarding the optimal timing and dosing of the hyperbaric oxygen treatment [81–83].

### 9.3. Hypothermia

Hypothermia has been implicated as an aggravating factor in critically ill patients, including those with serious burns [84, 85]. On the other hand, therapeutic hypothermia has been proposed to be beneficial in an array of human pathologies including cardiac arrest, stroke, traumatic brain and spinal cord injury, and hemorrhagic shock. Burn depth progression is multifactorial but inflammation plays a significant role. Systemic hypothermia decreased burn depth progression in a rodent model and upregulation of skin-protective genes and downregulation of detrimental tissue remodeling genes by hypothermia may contribute to its beneficial effects. Rizzo et al. [86] applied moderate hypothermia in the range of 31–33°C for 4 h both immediately after burn injury and in a delayed fashion, beginning 2 h after thermal injury model in rats. Immediate hypothermia decreased burn depth progression at 6 h after injury, and this protective effect was sustained for at least 24 h. Increased expression of several skin-protective genes and decreased expression of tissue remodeling genes were discovered in the skin biopsy samples of rats subjected to immediate hypothermia.

### 9.4. Analgesia

Peripherally active opioids have anti-inflammatory effects and can modulate wound healing. Local opioid application is being used for pain reduction in patients with inflammatory lesions such as burns [87]. Opioid agonists can attenuate the excitability of primary afferent neurons and block the release of proinflammatory neuropeptides from central and peripheral terminals [88]. The discovery that opioid receptors on sensory nerves are upregulated during subcutaneous inflammation prompted the search for endogenous ligands within inflamed tissue. Opioids can interfere at several different stages in the inflammatory process, both in somatic and visceral inflammations [89–91]. 

## 10. Conclusions

Burns are unique among acute injuries in the progressive nature of tissue necrosis and possible serious complications following the initial trauma, such as SIRS and severe metabolic imbalance. This intense instability in homeostasis may result in multiple organ failure and death. 

Current treatment of burn shock includes prompt fluid resuscitation and early burn wound excision. However, aggressive crystalloid-based resuscitation strategies are also associated with inflammatory mediator dysfunction. Hence, the patient's care team must always be careful to restrict the supply of liquid to only what is necessary to rescue the hypovolemic shock. Early excision of burns plays an important role in attenuation of SIRS as it can decrease the release of inflammatory mediators and the bacterial colonization of wounds. Over the past twenty years, since the introduction of early excision and grafting in our burn unit, we have observed a reduction in the mortality rate from 12% to less than 5%.

Currently, alternative therapies are emerging that seek to modulate the complex systemic inflammation in burns. We support the discovery of new therapeutic options that can modulate the production of inflammatory cytokines which we believe will lead to improved treatment of burns. Inherently, the knowledge of the balance between pro- and anti-inflammatory cytokines is of fundamental importance and this also becomes a challenge to be overcome in the future. The aim of this paper is to comprehensively review current therapies available which can inhibit the inflammatory response to burns. 

## Figures and Tables

**Table 1:** Mortality rate in the burn unit of HC-FMRP-USP-Brazil from 1992 to 2012.

Year	Survivors	Nonsurvivors	Total no. of cases	Mortality rate (%)
1992	98	14	112	12.5
1993	107	13	120	10.8
1994	83	8	91	8.8
1995	97	6	103	5.8
1996	142	5	147	3.4
1997	123	9	132	6.8
1998	141	9	150	6.0
1999	151	6	157	3.8
2000	144	6	150	4.0
2001	139	4	143	2.8
2002	142	5	147	3.4
2003	142	1	143	0.7
2004	118	11	129	8.5
2005	161	5	166	3.0
2006	178	8	186	4.3
2007	197	8	205	3.9
2008	246	10	256	3.9
2009	245	5	250	2.0
2010	217	10	227	4.4
2011	193	7	200	3.5
2012	208	3	211	1.4

## Abbreviations

SIRS: Systemic inflammatory response syndrome
BSA: Body surface area
mL: Milliliter
kg: Kilogram
LPC: Lipid protein complex
TNF-*α*: Tumor necrosis factor alpha
IL-1b: Interleukin-1b
IL-6: Interleukin-6
IL-8: Interleukin-8
IL-10: Interleukin-10
IFN-*γ*: Interferon-gamma
EPC: Endothelial progenitor cells
HC-FMRP-USP: Hospital das Clínicas da Faculdade de Medicina de Ribeirão Preto da Universidadede São Paulo-Brazil;
Th1: (T-helper-1) cytokines
APC: Activated protein C
RBP: Retinol binding protein
IR: Insulin resistance
bFGF: basic fibroblast growth factor
TGF*α*: Transforming growth factor alpha
MB: Methylene blue
VS: Vasoplegic syndrome
NO: Nitric oxide
cGMP: Cyclic guanosine monophosphate
sGC: Soluble guanylatecyclase
NOS: Nitric oxide synthase
DNA: Deoxyribonucleic acid
TGF-*β*1: Transforming growth factor beta-1
DNAse: Deoxyribonuclease
MSC: Mesenchymal stem cell
Akt1-V: Murine thymoma viral oncogene homolog 1
°C: Celsius degree
h: Hour.

## References

[1] Oppeltz RF, Zhang Q, Rani M, Sasaki JR, Schwacha MG (2010). Increased expression of cardiac IL-17 after burn. *Journal of Inflammation*.

[2] Ipaktchi K, Arbabi S (2006). Advances in burn critical care. *Critical Care Medicine*.

[3] Soejima A, Miyake N, Matsuzawa N (1998). Clinical characterization of acute renal failure in multiple organ dysfunction syndrome. *Clinical and Experimental Nephrology*.

[4] Macintire DK, Bellhorn TL (2002). Bacterial translocation: clinical implications and prevention. *Veterinary Clinics of North America—Small Animal Practice*.

[5] Meshulam-Derazon S, Nachumovsky S, Ad-El D, Sulkes J, Hauben DJ (2006). Prediction of morbidity and mortality on admission to a burn unit. *Plastic and Reconstructive Surgery*.

[6] Ashworth HL, Cubison TC, Gilbert PM, Sim KM (2001). Treatment before transfer: the patient with burns. *Emergency Medicine Journal*.

[7] Saffle JR (2007). The phenomenon of “fluid creep” in acute burn resuscitation. *Journal of Burn Care and Research*.

[8] Pruitt BA (2000). Protection from excessive resuscitation:‘pushing the pendulum back’. *Journal of Trauma*.

[9] Faraklas I, Cochran A, Saffle J (2012). Review of a fluid resuscitation protocol: “fluid creep” is not due to nursing error. *Journal of Burn Care and Research*.

[10] Cotton BA, Guy JS, Morris JA, Abumrad NN (2006). The cellular, metabolic, and systemic consequences of aggressive fluid resuscitation strategies. *Shock*.

[11] Friedrich JB, Sullivan SR, Engrav LH (2004). Is supra-Baxter resuscitation in burn patients a new phenomenon?. *Burns*.

[12] Ivy ME, Atweh NA, Palmer J, Possenti PP, Pineau M, D’Aiuto M (2000). Intra-abdominal hypertension and abdominal compartment syndrome in burn patients. *Journal of Trauma*.

[13] Zhang JP, Xiang F, Tong DL (2012). Comparative study on the effect of restrictive fluid management strategy on the early pulmonary function of patients with severe burn. *Chinese Journal of Burns*.

[14] Garner JP, Heppell PSJ (2005). Cerium nitrate in the management of burns. *Burns*.

[15] Schoenenberger GA, Bauer UR, Cueni LB, Eppenberger U, Allgöwer M (1971). Isolation and characterization of a cutaneous lipoprotein with lethal effects produced by thermal energy in mouse skin. *Biochemical and Biophysical Research Communications*.

[16] Molloy RG, O'Riordain M, Holtzeimer R (1993). Mechanisms of increased tumor necrosis factor production after thermal injury. Altered sensitivity to PG2 and immune modulation by indomethacin. *Journal of Immunology*.

[17] Ogle CK, Mao JX, Wu JZ, Ogle JD, Alexander JW (1994). The 1994 Lindberg Award: the production of tumor necrosis factor, interleukin-1, interleukin-6, and prostaglandin E2 by isolated enterocytes and gut macrophages: effect of lipopolysaccharide and thermal injury. *Journal of Burn Care and Rehabilitation*.

[18] Rumbaugh KP, Colmer JA, Griswold JA, Hamood AN (2001). The effects of infection of thermal injury by Pseudomonas aeruginosa PAO1 on the murine cytokine response. *Cytokine*.

[19] Accardo Palumbo A, Forte GI, Pileri D (2012). Analysis of IL-6, IL-10 and IL-17 genetic polymorphisms as risk factors for sepsis development in burned patients. *Burns*.

[20] Sakallioglu AE, Basaran O, Karakayali H (2006). Interactions of systemic immune response and local wound healing in different burn depths: an experimental study on rats. *Journal of Burn Care and Research*.

[21] Ong YS, Samuel M, Song C (2006). Meta-analysis of early excision of burns. *Burns*.

[22] Janzekovic Z (1970). A new concept in the early excision and immediate grafting of burns. *Journal of Trauma*.

[23] Wang ZT, Yao YM, Sheng ZY (2004). Effects of escharectomy during shock stage on tissue high mobility group box-1 expression and balance of pro-/anti-inflammatory response in rats after severe thermal injury. *Chinese Journal of Surgery*.

[24] Chen XL, Xia ZF, Wei HF (2011). Escharectomy and allografting during shock stage reduces insulin resistance induced by major burn. *Journal of Burn Care and Research*.

[25] Foresta C, Schipilliti M, de Toni L (2011). Blood levels, apoptosis, and homing of the endothelial progenitor cells after skin burns and escharectomy. *Journal of Trauma*.

[26] Han TH, Lee SY, Kwon JE, Kwak IS, Kim KM (2004). The limited immunomodulatory effects of escharectomy on the kinetics of endotoxin, cytokines, and adhesion molecules in major burns. *Mediators of Inflammation*.

[27] Sun LT, Friedrich E, Heuslein JL (2012). Reduction of burn progression with topical delivery of (antitumor necrosis factor-*α*)-hyaluronic acid conjugates. *Wound Repair and Regeneration*.

[28] Sherwood ER, Varma TK, Fram RY, Lin CY, Koutrouvelis AP, Toliver-Kinsky TE (2001). Glucan phosphate potentiates endotoxin-induced interferon-*γ* expression in immunocompetent mice, but attenuates induction of endotoxin tolerance. *Clinical Science*.

[29] Williams DL, McNamee RB, Jones EL (1991). A method for the solubilization of a (1→3)-*β*-D-glucan isolated from Saccharomyces cerevisiae. *Carbohydrate Research*.

[30] Lyuksutova OI, Murphey ED, Toliver-Kinsky TE (2005). Glucan phosphate treatment attenuates burn-induced inflammation and improves resistance to Pseudomonas aeruginosa burn wound infection. *Shock*.

[31] Cao YZ, Tu YY, Chen X (2012). Protective effect of ulinastatin against murine models of sepsis: inhibition of TNF-alpha and IL-6 and augmentation of IL-10 and IL-13. *Experimental and Toxicologic Pathology*.

[32] Luo HM, Hu S, Zhou GY (2012). The effects of ulinastatin on systemic inflammation, visceral vasopermeability and tissue water content in rats with scald injury. *Burns*.

[33] Rehberg S, Enkhbaatar P, Cox RA, Traber DL (2012). Coagulopathy after burn and smoke inhalation injury: the evidence is there, let’s take advantage of it!. *The Journal of Trauma and Acute Care Surgery*.

[34] Lavrentieva A (2013). Replacement of specific coagulation factors in patients with burn: a review. *Burns*.

[35] Lavrentieva A, Kontakiotis T, Bitzani M (2008). Early coagulation disorders after severe burn injury: impact on mortality. *Intensive Care Medicine*.

[36] Lippi G, Ippolito L, Cervellin G (2010). Disseminated intravascular coagulation in burn injury. *Seminars in Thrombosis and Hemostasis*.

[37] García-Avello A, Lorente JA, Cesar-Perez J (1998). Degree of hypercoagulability and hyperfibrinolysis is related to organ failure and prognosis after burn trauma. *Thrombosis Research*.

[38] Niedermayr M, Schramm W, Kamolz L (2007). Antithrombin deficiency and its relationship to severe burns. *Burns*.

[39] Bartlett RH, Fong SV, Maruggo G, Hardeman T, Anderson V (1981). Coagulation and platelets changes after thermal injury in man. *Burns*.

[40] Kowal-Vern A, Gamelli RL, Walenga JM (1992). The effect of burn wound size on hemostasis: a correlation of the hemostatic changes to the clinical state. *Journal of Trauma*.

[41] Nisanci M, Eski M, Sahin I, Ilgan S, Isik S (2010). Saving the zone of stasis in burns with activated protein C: an experimental study in rats. *Burns*.

[42] Walker FJ, Sexton PW, Esmon CT (1979). The inhibition of blood coagulation by activated protein C through the selective inactivation of activated factor V.. *Biochimica et Biophysica Acta*.

[43] Hoffman M (2003). A cell-based model of coagulation and the role of factor VIIa. *Blood Reviews*.

[44] Fan J, Meng Q, Guo G (2010). Effects of early enteral nutrition supplemented with arginine on intestinal mucosal immunity in severely burned mice. *Clinical Nutrition*.

[45] Garcia de Lorenzo y Mateos A, Caparros Fernandez de Aguilar A, Blesa Malpica A (2000). Multiple trauma and burns. *Nutrición Hospitalaria*.

[46] Kraft R, Herndon DN, Kulp GA (2011). Retinol binding protein: marker for insulin resistance and inflammation postburn?. *Journal of Parenteral and Enteral Nutrition*.

[47] Jeschke MG, Klein D, Herndon DN (2004). Insulin treatment improves the systemic inflammatory reaction to severe trauma. *Annals of Surgery*.

[48] Orman MA, Nguyen TT, Ierapetritou MG, Berthiaume F, Androulakis IP (2011). Comparison of the cytokine and chemokine dynamics of the early inflammatory response in models of burn injury and infection. *Cytokine*.

[49] Lord GM, Matarese G, Howard JK, Baker RJ, Bloom SR, Lechler RI (1998). Leptin modulates the T-cell immune response and reverses starvation-induced immunosuppression. *Nature*.

[50] Fantuzzi G, Faggioni R (2000). Leptin in the regulation of immunity, inflammation, and hematopoiesis. *Journal of Leukocyte Biology*.

[51] Caldefie-Chezet F, Poulin A, Tridon A, Sion B, Vasson MP (2001). Leptin: a potential regulator of polymorphonuclear neutrophil bactericidal action?. *Journal of Leukocyte Biology*.

[52] Faggioni R, Feingold KR, Grunfeld C (2001). Leptin regulation of the immune response and the immunodeficiency of malnutrition. *FASEB Journal*.

[53] Çakir B, Çevik H, Contuk G, Ercan F, Ekşioğlu-Demiralp E, Yeğen BÇ (2005). Leptin ameliorates burn-induced multiple organ damage and modulates postburn immune response in rats. *Regulatory Peptides*.

[54] Abdel-Hafez NM, Saleh Hassan Y, El-Metwally TH (2007). A study on biomarkers, cytokines, and growth factors in children with burn injuries. *Annuals of Burns Fire Disasters*.

[55] Jeschke MG, William FN, Finnerty CC (2012). The effect of ketoconazole on post-burn inflammation, hypermetabolism and clinical outcomes. *PLoS One*.

[56] LaLonde C, Nayak U, Hennigan J, Demling RH (1997). Excessive liver oxidant stress causes mortality in response to burn injury combined with endotoxin and is prevented with antioxidants. *Journal of Burn Care and Rehabilitation*.

[57] Berger MM, Shenkin A (2007). Trace element requirements in critically ill burned patients. *Journal of Trace Elements in Medicine and Biology*.

[58] Soneja A, Drews M, Malinski T (2005). Role of nitric oxide, nitroxidative and oxidative stress in wound healing. *Pharmacological Reports*.

[59] Kehrer JP (1993). Free radicals as mediators of tissue injury and disease. *Critical Reviews in Toxicology*.

[60] Berger MM, Binnert C, Chiolero RL (2007). Trace element supplementation after major burns increases burned skin trace element concentrations and modulates local protein metabolism but not whole-body substrate metabolism. *The American Journal of Clinical Nutrition*.

[61] Barbosa E, Faintuch J, MacHado Moreira EA (2009). Supplementation of vitamin E, vitamin C, and zinc attenuates oxidative stress in burned children: a randomized, double-blind, placebo-controlled pilot study. *Journal of Burn Care and Research*.

[62] Tanaka H, Matsuda T, Miyagantani Y, Yukioka T, Matsuda H, Shimazaki S (2000). Reduction of resuscitation fluid volumes in severely burned patients using ascorbic acid administration: a randomized, prospective study. *Archives of Surgery*.

[63] Csontos C, Rezman B, Foldi V (2012). Effect of N-acetylcysteine treatment on oxidative stress and inflammation after severe burn. *Burns*.

[64] Vinha PP, Martinez EZ, Vannucchi H (2013). Effect of acute thermal injury in status of serum vitamins, inflammatory and oxidative stress markers: preliminary data. *Journal of Burn Care and Research*.

[65] Wang J, Huang CJ, Chow CK (1996). Red cell vitamin E and oxidative damage: a dual role of reducing agents. *Free Radical Research*.

[66] Buege JA, Aust SD (1978). Microsomal lipid peroxidation. *Methods in Enzymology*.

[67] Farina JA, Celotto AC, da Silva MF, Evora PR (2012). Guanylatecyclase inhibition by methylene blue as an option in the treatment of vasoplegia after a severe burn. A medical hypothesis. *Medical Science Monitor*.

[68] Jaskille AD, Jeng JC, Jordan MH (2008). Methylene blue in the treatment of vasoplegia following severe burns. *Journal of Burn Care and Research*.

[69] Inoue H, Ando K, Wakisaka N, Matsuzaki KI, Aihara M, Kumagai N (2001). Effects of nitric oxide synthase inhibitors on vascular hyperpermeability with thermal injury in mice. *Nitric Oxide—Biology and Chemistry*.

[70] Evora PRB, Rodrigues AJ, Vicente WVDA (2007). Is the cyclic GMP system underestimated by intensive care and emergency teams?. *Medical Hypotheses*.

[71] Sterner JB, Zanders TB, Morris MJ, Cancio LC (2009). Inflammatory mediators in smoke inhalation injury. *Inflammation and Allergy—Drug Targets*.

[72] Joyner BL, Jones SW, Cairns BA (2013). DNA and inflammatory mediators in bronchoalveolar lavage fluid from children with acute inhalational injuries. *Journal of Burn Care and Research*.

[73] Kirchner KK, Wagener JS, Khan TZ, Copenhaver SC, Accurso FJ (1996). Increased DNA levels in bronchoalveolar lavage fluid obtained from infants with cystic fibrosis. *American Journal of Respiratory and Critical Care Medicine*.

[74] Paul K, Rietschel E, Ballmann M (2004). Effect of treatment with dornase alpha on airway inflammation in patients with cystic fibrosis. *American Journal of Respiratory and Critical Care Medicine*.

[75] Ratjen F, Paul K, van Koningsbruggen S, Breitenstein S, Rietschel E, Nikolaizik W (2005). DNA concentrations in BAL fluid of cystic fibrosis patients with early lung disease: influence of treatment with dornase alpha. *Pediatric Pulmonology*.

[76] Zhu F, Guo GH, Chen W, Peng Y, Xing JJ, Wang NY (2010). Effect of bone marrow-derived mesenchymal stem cells transplantation on the inflammatory response and lung injury in rabbit with inhalation injury. *Chinese Journal of Burns*.

[77] Butler KL, Goverman J, Ma H (2010). Stem cells and burns: review and therapeutic implications. *Journal of Burn Care and Research*.

[78] Huang L, Burd A (2012). An update review of stem cell applications in burns and wound care. *Indian Journal of Plastic Surgery*.

[79] Yagi H, Soto-Gutierrez A, Kitagawa Y, Tilles AW, Tompkins RG, Yarmush ML (2010). Bone marrow mesenchymal stromal cells attenuate organ injury induced by LPS and burn. *Cell Transplantation*.

[80] Tayyar SC, Ozalp B, Durgun M (2012). The effect of hyperbaric oxygen treatment on the healing of burn wounds in nicotinized and nonnicotinized rats. *Journal of Burn Care and Research*.

[81] Türkaslan T, Yogun N, Çimşit M, Solakoglu S, Ozdemir C, Ozsoy Z (2010). Is HBOT treatment effective in recovering zone of stasis? An experimental immunohistochemical study. *Burns*.

[82] Shoshani O, Shupak A, Barak A (1998). Hyperbaric oxygen therapy for deep second degree burns: an experimental study in the guinea pig. *British Journal of Plastic Surgery*.

[83] Bilic I, Petri NM, Bezic J (2005). Effects of hyperbaric oxygen therapy on experimental burn wound healing in rats: a randomized controlled study. *Undersea and Hyperbaric Medicine*.

[84] White CE, Renz EM (2008). Advances in surgical care: management of severe burn injury. *Critical Care Medicine*.

[85] Moffatt SE (2012). Hypothermia in trauma. *Emergency Medicine Journal*.

[86] Rizzo JA, Burgess P, Cartie RJ, Prasad BM (2013). Moderate systemic hypothermia decreases burn depth progression. *Burns*.

[87] Stein C, Küchler S (2012). Non-analgesic effects of opioids: peripheral opioid effects on inflammation and wound healing. *Current Pharmaceutical Design*.

[88] Stein C, Hassan AHS, Przewlocki R, Gramsch C, Peter K, Herz A (1990). Opioids from immunocytes interact with receptors on sensory nerves to inhibit nociception in inflammation. *Proceedings of the National Academy of Sciences of the United States of America*.

[89] Tegeder I, Geisslinger G (2004). Opioids as modulators of cell death and survival—unraveling mechanisms and revealing new indications. *Pharmacological Reviews*.

[90] Rittner HL, Machelska H, Stein C (2005). Leukocytes in the regulation of pain and analgesia. *Journal of Leukocyte Biology*.

[91] Chen YL, Law PY, Loh HH (2008). The other side of the opioid story: modulation of cell growth and survival signaling. *Current Medicinal Chemistry*.

